# Hybrid Dynamic Traffic Model for Freeway Flow Analysis Using a Switched Reduced-Order Unknown-Input State Observer

**DOI:** 10.3390/s20061609

**Published:** 2020-03-13

**Authors:** Yuqi Guo, Bin Li, Matthew Daniel Christie, Zongzhi Li, Miguel Angel Sotelo, Yulin Ma, Dongmei Liu, Zhixiong Li

**Affiliations:** 1 Research Institute of Highway, Ministry of Transport of China, Beijing 100088, China; gyq@itsc.cn (Y.G.); libin@itsc.cn (B.L.); ldm@itsc.cn (D.L.); 2 School of Mechanical, Materials, Mechatronic and Biomedical Engineering, University of Wollongong, Wollongong, NSW 2522, Australia; mdc769@uowmail.edu.au; 3 Department of Civil, Architectural and Environmental Engineering, Illinois Institute of Technology, Chicago, IL 60616, USA; lizz@iit.edu; 4 Department of Computer Engineering, University of Alcalá, 28801 Alcalá de Henares (Madrid), Spain; miguel.sotelo@uah.es; 5 Suzhou Automotive Research Institute, Tsinghua University, Suzhou 215134, China; zhixiong_li@uow.edu.au

**Keywords:** urban freeway, hybrid dynamic system, state transition, unknown inputs observer, vehicle density

## Abstract

This paper introduces a new methodology for reconstructing vehicle densities of freeway segments by utilizing the limited data collected by traffic-counting sensors and developing a macroscopic traffic stream model formulated as a switched reduced-order state observer design problem with unknown or partially known inputs. Specifically, the traffic network is modeled as a hybrid dynamic system in a state space that incorporates unknown inputs. For freeway segments with traffic-counting sensors installed, vehicle densities are directly computed using field traffic count data. A reduced-order state observer is designed to analyze traffic state transitions for freeway segments without field traffic count data to indirectly estimate the vehicle densities for each freeway segment. A simulation-based experiment is performed applying the methodology and using data of a segment of Beijing Jingtong freeway in Beijing, China. The model execution results are compared with the field data associated with the same freeway segment, and highly consistent results are achieved. The proposed methodology is expected to be adopted by traffic engineers to evaluate freeway operations and develop effective management strategies.

## 1. Introduction

The estimation of vehicle densities on highway segments has been of considerable interest in recent decades. Research has been underway in developing practical methods in the context of the macroscopic traffic flow dynamic model for vehicle density estimation using different types of estimators. In particular, the state observer method [1,2] has been rapidly adopted by researchers and practitioners for traffic state estimation. In a study [3], traffic state was estimated by using an adaptive observer. Based on the cell transmission model, a centralized observer was considered in another work [4], and the estimation was further improved in a subsequent study [5]. In reference [6], the switched distributed observer was studied using the consensus theory. Based on the piecewise affine (PWA) system model of traffic network, various switched-state observers were also designed, such as the centralized observer [7,8,9,10,11], decentralized observer [8], and distributed observer [9,10], to estimate vehicle densities associated with segments of highways with different functional classifications.

It should be noted that the above-mentioned state observers were designed based on the ideal traffic flow dynamic model. That is, both the system disturbance and the measurement noise were not taken into consideration in the modeling procedure, and all the inputs were treated as measurable signals. Hence, these types of observers are termed as known-inputs state observers. As a practical matter, disturbances, especially unknown input signals, cannot be ignored in actual traffic networks. Otherwise, the proposed model could not reflect the real traffic flow transmission rule.

With advancements of sensing and positioning technologies, measurement accuracy and precision of traffic sensors have significantly improved, leading to the possibility of ignoring measurement noises. However, unknown inputs must be explicitly accounted for owing to their impacts on the predictability of the proposed models. The existing literature largely considers dynamic models with unknown inputs, while measurement noises are ignored. In the context of unknown-input observer models, dynamic models are classified as linear and nonlinear systems [12,13,14,15,16,17,18,19,20,21,22,23,24], continuous and discrete systems [19,21,25,26], and time-varying and time-invariant systems [27,28]. For instance, the unknown-input observer was studied based on the optimal data fusion approach [29]. Both full-order and reduced-order observers for continuous dynamical systems with unknown inputs were investigated [29]. A design strategy of the nonlinear unknown-input state observer for a nonlinear system was reported [30]. A novel full-order unknown-input observer for the continuous system along with the existence of the necessary conditions was proposed [31]. Additional unknown-input observers for different types of systems were also reported [32,33,34,35].

In addition, as an effective and practical tool, deep learning methods are also popular in estimating traffic flows. In a study [36], spatio-temporal factors were considered in traffic prediction, and a multi-attention network was proposed to predict traffic conditions at different locations on a road network graph. In order to deeply capture the high-order spatial–temporal correlations among the road links, Zhang et al. [37] performed a road network-level prediction using a network-scale deep traffic prediction model, called TrafficGAN, where the generative adversarial nets were utilized to predict traffic flows under an adversarial learning framework. In another study [38], a deep learning framework was presented to solve the traffic forecasting problem; the traffic flow was modelled as a diffusion process on a direct graph, and a diffusion convolutional recurrent neural network was introduced to predict the traffic flow.

Estimation of traffic flow density is the basis of many transportation applications, such as route planning and vehicle routing [39,40,41]. For example, based on the estimation of the traffic flow density, the distribution of traffic congestion in a road network was identified, and then, alternative routes were assigned for selected vehicles to avoid congested roads [39]. Typically, traffic network can be modeled as a hybrid dynamic system by means of multi-mode switching [11]. However, most of the existing state observers were designed on the basis of the ideal traffic flow dynamic model, where both the system disturbance and the measurement noise were not taken into consideration in the modeling procedure. Especially, the inputs were treated as measurable signals and were known in advance. Hence, these types of observers are termed known-input state observers. From a practical viewpoint, system disturbance and measurement noise always exist in the actual traffic flow network, and these input signals are often unknown but cannot be ignored. In order to estimate vehicle density for real-world freeway segments, a traffic flow model should include system disturbance and measurement noise. However, few studies have systematically addressed the vehicle density estimation problem with unknown inputs. It is crucial to explore a more effective way to solve the design challenge of the unknown-input state observer on the basis of the hybrid dynamic traffic network model. To this avail, this paper introduces a switched unknown-input state observer to reconstruct vehicle densities of segments of a highway system which maintains field data measurements by using traffic sensors for only a fraction of segments and limited known inputs. Compared with other existing methods, the proposed dynamic model has the following advantages: (1) it is more realistic in representing the actual traffic networks and (2) the actual vehicle densities can be readily reconstructed using the unknown-input state observer.

The remainder of the paper is organized as follows. Section 2 provides the background information on the traffic flow dynamic model and elaborates the proposed observer model with unknown inputs state. Section 3 evaluates the proposed model using real-world traffic data. Section 4 summarizes the findings of this study.

## 2. Proposed Method

**Figure 1:**
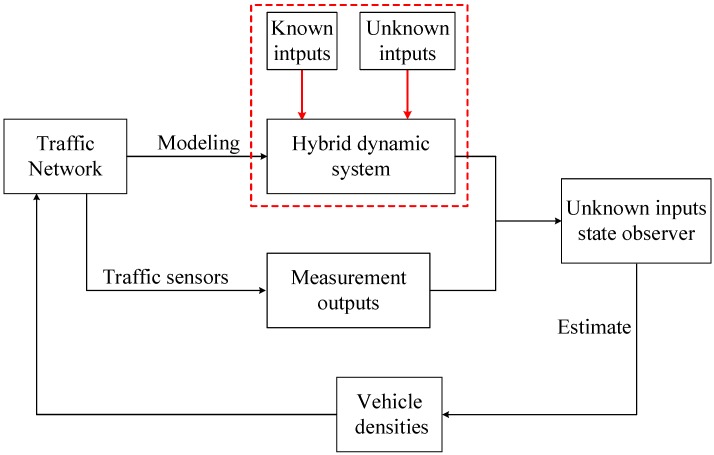
An overview of the proposed method.

An overview of the proposed traffic density estimation method is given in Figure 1. The details of constructing the predictive model are described in the following sub-sections.

### 2.1. Hybrid Dynamic System

This section first reviews the hybrid dynamic traffic flow model that combines the dynamic graph hybrid automata with the cell transmission model (CTM) and then briefly describes the problem of vehicle density estimation. The hybrid dynamic traffic flow model is described by

(1) $$\{\begin{array}{l} x\left(t+1\right)=A_{\sigma \left(t\right)}x\left(t\right)+B_{\sigma \left(t\right)}u\left(t\right)\\ y\left(t\right)=Cx\left(t\right) \end{array}$$

where $x={\left[\rho_{1},\cdots ,\rho_{n}\right]}^{T}\in R^{n}$ represents the vehicle density vector, $u\in R^{m}$ is the input vector, $y\in R^{q}$ is the measured output vector, $A_{\sigma }$, $B_{\sigma }$, and C are the system matrix, the input matrix, and the output matrix, respectively, σ:[0,+∞)→{1,2,⋯,s} is the switching function that maps the index time stage into an index set {1,2,⋯,s}, and each index corresponds to a different mode of the system.

Based on the dynamic model shown above, different types of state observers can be designed to estimate the vehicle densities of a traffic stream [7,8,9,10,11]. However, it is difficult to apply this method to a real-world traffic network. This is because all sources of disturbances associated with unknown-input signals and measurement errors are ignored in the modeling process, making it difficult to apply the actual traffic flow transmission rule. Further, the exclusion of measurement errors in the design of state observers renders the estimated data incapable of reflecting the real traffic states. Therefore, disturbances need to be included in the base model, which creates the augmented model

(2) $$\{\begin{array}{l} x\left(t+1\right)=A_{\sigma \left(t\right)}x\left(t\right)+B_{\sigma \left(t\right)}u\left(t\right)+D_{\sigma \left(t\right)}v\left(t\right)\\ y\left(t\right)=Cx\left(t\right) \end{array}$$

where $v\in R^{p}$ is the unknown-input signal, $u\in R^{m}$ is the known-input signal, $D_{\sigma }\in R^{p\times n}$ is the known noise matrix with appropriate dimensions. The others are the same as in the model described by (1).

**Remark** **1.** *With the development of sensor and positioning technologies, the accuracy and precision of both mobile and fixed traffic detectors have been improving. The influence of measurement errors can be marginally neglected in most cases. However, system noise cannot be completely eliminated. As such, the augmented dynamic model ignores the measurement errors, while it incorporates unknown inputs to capture the system noise.*

The augmented model deals with situations where traffic data are partially available for some highway segments equipped with sensors. For the remaining highway segments without traffic sensors, a state observer needs to be designed to reconstruct the traffic states. To reduce the structural complexity of the traffic state observer, it is advantageous to design a reduced-order state observer.

### 2.2. Unknown-Input State Observer

This section introduces the design of the reduced-order unknown-input state observer for the augmented dynamic traffic model of vehicle density estimation. The essence of the state observer design is to accurately reconstruct vehicle densities $\hat{x}$ and estimate the unknown inputs $\hat{v}$ for the system {u,v,y,A,B,C,D}, such that the following conditions are satisfied:

(3) $$\{\begin{array}{l} e(t)=x(t)-\hat{x}(t)\to 0\\ \tilde{v}(t)=v(t)-\hat{v}(t)\to 0 \end{array}$$

where x is the vehicle density vector, $\hat{x}$ is the estimated density vector, v is the input vector, $\hat{v}$ is the estimated input vector, e is the estimation error between the actual and the estimated vehicle densities, and $\tilde{v}$ is the estimation error between the real and the estimated inputs.

**Definition** **1.** ***Unknown-Input State Observer*** *: A state observer is defined as an unknown-input observer if its estimation error approaches zero asymptotically, regardless of the presence of unknown inputs or disturbances in a dynamic system.*

**Definition** **2.** ***Switched Unknown-Input State Observer*** *: A state observer is termed a switched unknown-input observer for a dynamic system if, and only if, its state estimation error system is asymptotically stable for any switching sequence, regardless of the existence of unknown inputs in the system.*

As the preparation for the unknown-input state observer design, the following three assumptions were made:

(i) $\mathrm{rank}D_{\sigma }=p$ and rankC=q.
(ii) The pair $\left(A_{\sigma },C\right)$ is observable or detectable.
(iii) $q\geq p,\;\mathrm{rank}\left(CD_{\sigma }\right)=p$.

**Remark** **2.** *The above assumptions imply that the matrices* C *and* $D_{\sigma }$ *are full row rank and full column rank, respectively. These characteristics can always be met by optimizing the configuration of the matrix* C *and redefining the noise matrix* $D_{\sigma }$ *.*

Since rankC=q, $R\in R^{\left(n-q\right)\times n}$ can be arbitrarily chosen, and the following transformation matrix $P\in R^{n\times n}$ is nonsingular.

(4) $$P≜\left[\begin{array}{c} C\\ R \end{array}\right]$$

The inverse matrix of P is denoted as

(5) $$Q≜P^{-1}={\left[\begin{array}{c} C\\ R \end{array}\right]}^{-1}=\left[\begin{array}{cc} Q_{1} & Q_{2} \end{array}\right]$$

The matrix *R* is not unique but needs to be chosen to ensure that the matrix *P* is invertible. By using linear nonsingular transformation, the corresponding matrices can be rewritten as follows

(6) $$\{\begin{array}{l} \bar{A}=QAQ^{-1}=\left[\begin{array}{cc} {\bar{A}}_{11} & {\bar{A}}_{12}\\ {\bar{A}}_{21} & {\bar{A}}_{22} \end{array}\right]\\ \bar{B}=QB=\left[\begin{array}{c} {\bar{B}}_{1}\\ {\bar{B}}_{2} \end{array}\right]\\ \bar{C}=CQ=\left[\begin{array}{cc} I_{q} & 0 \end{array}\right]\\ \bar{D}=QD=\left[\begin{array}{c} {\bar{D}}_{1}\\ {\bar{D}}_{2} \end{array}\right] \end{array}$$

Subsequently, by using the transformation $\bar{x}=Q^{-1}x$ and $y=CQ\bar{x}$, the dynamic system (2) can be re-constructed with the following specification:

(7) $$\{\begin{array}{l} \begin{array}{l} \left[\begin{array}{c} {\bar{x}}_{1}\left(t+1\right)\\ {\bar{x}}_{2}\left(t+1\right) \end{array}\right]={\left[\begin{array}{cc} {\bar{A}}_{11} & {\bar{A}}_{12}\\ {\bar{A}}_{21} & {\bar{A}}_{22} \end{array}\right]}_{\sigma \left(t\right)}\left[\begin{array}{c} {\bar{x}}_{1}\left(t\right)\\ {\bar{x}}_{2}\left(t\right) \end{array}\right]+{\left[\begin{array}{c} {\bar{B}}_{1}\\ {\bar{B}}_{2} \end{array}\right]}_{\sigma \left(t\right)}u\left(t\right)+{\left[\begin{array}{c} {\bar{D}}_{1}\\ {\bar{D}}_{2} \end{array}\right]}_{\sigma \left(t\right)}v\left(t\right) \end{array}\\ y\left(t\right)=\left[\begin{array}{cc} I_{q} & 0 \end{array}\right]\left[\begin{array}{c} {\bar{x}}_{1}\left(t\right)\\ {\bar{x}}_{2}\left(t\right) \end{array}\right] \end{array}$$

where ${\bar{x}}_{1}=y\in R^{q}$, ${\bar{x}}_{2}\in R^{n-q}$.

The above analysis shows that vehicle densities ${\bar{x}}_{1}$ can be obtained by the measurement output y, while only partial vehicle densities ${\bar{x}}_{2}$ need an estimate. Hence, the reduced-order state observer needs to be designed to complete the density reconstruction.

**Remark** **3.** *For segments of a highway network with field traffic-counting sensors installed, traffic states can be directly assessed by traffic data collected by the sensors. Conversely, for highway segments without field traffic-counting sensors, a state observer needs to be designed to estimate the traffic states. The joint use of field traffic count data and traffic state estimates by the state observer could help derive highly accurate and precise values of vehicle densities. As such, vehicle density estimation boils down to the design of an effective state observer. Practically, designing a reduced-order state observer becomes the key to solving the vehicle density estimation problem.*

Based on Remark 3, the dynamic system (7) can be formulated as the following:

(8) $$\{\begin{array}{l} {\bar{x}}_{2}\left(t+1\right)={\bar{A}}_{22_{\sigma \left(t\right)}}{\bar{x}}_{2}\left(t\right)+{\bar{A}}_{21_{\sigma \left(t\right)}}y\left(t\right)+{\bar{B}}_{2}u\left(t\right)+{\bar{D}}_{2_{\sigma \left(t\right)}}v\left(t\right)\\ y(t+1)-{\bar{A}}_{11_{\sigma \left(t\right)}}y\left(t\right)={\bar{A}}_{12_{\sigma \left(t\right)}}{\bar{x}}_{2}\left(t\right)+{\bar{B}}_{1}u\left(t\right)+{\bar{D}}_{1_{\sigma \left(t\right)}}v\left(t\right) \end{array}$$

**Theorem** **1.** *In the presence of an invertible matrix* P *, such that Equation (8) is satisfied, and the pair* $\left(A_{\sigma },C\right)$ *is observable or detectable, there must exist a reduced-order state observer in the form (9), such that the vehicle densities of the system (2) can be estimated.*

The reduced-order state observer can be obtained by

(9) $$\{\begin{array}{l} z\left(t+1\right)=F_{\sigma }z\left(t\right)+G_{\sigma }y\left(t\right)+H_{\sigma }u\left(t\right)\\ \hat{x}\left(t\right)=Q_{1}y\left(t\right)+Q_{2}\left(z+L_{\sigma }y\right) \end{array}$$

The proof for the state observer begins with (8), which can be further rewritten as

(10) $$\{\begin{array}{l} {\bar{x}}_{2}\left(t+1\right)={\bar{A}}_{22_{\sigma \left(t\right)}}{\bar{x}}_{2}\left(t\right)+\left[{\bar{A}}_{21_{\sigma \left(t\right)}}y\left(t\right)+{\bar{B}}_{2}u\left(t\right)+{\bar{D}}_{2_{\sigma \left(t\right)}}v\left(t\right)\right]\\ y(t+1)-{\bar{A}}_{11_{\sigma \left(t\right)}}y\left(t\right)-{\bar{B}}_{1}u\left(t\right)-{\bar{D}}_{1_{\sigma \left(t\right)}}v\left(t\right)={\bar{A}}_{12_{\sigma \left(t\right)}}{\bar{x}}_{2}\left(t\right) \end{array}$$

Based on the following equivalent substitution

(11) $$\{\begin{array}{l} \bar{u}\left(t\right)≜{\bar{A}}_{21_{\sigma \left(t\right)}}y\left(t\right)+{\bar{B}}_{2}u\left(t\right)+{\bar{D}}_{2_{\sigma \left(t\right)}}v\left(t\right)\\ w\left(t\right)≜y(t+1)-{\bar{A}}_{11_{\sigma \left(t\right)}}y\left(t\right)-{\bar{B}}_{1}u\left(t\right)-{\bar{D}}_{1_{\sigma \left(t\right)}}v\left(t\right) \end{array}$$

the following normative form is obtained:

(12) $$\{\begin{array}{l} {\bar{x}}_{2}\left(t+1\right)={\bar{A}}_{22_{\sigma \left(t\right)}}{\bar{x}}_{2}\left(t\right)+\bar{u}\left(t\right)\\ w\left(t\right)={\bar{A}}_{12_{\sigma \left(t\right)}}{\bar{x}}_{2}\left(t\right) \end{array}$$

It should be noted that observability or detectability of the pair $\left(A_{\sigma },C\right)$ implies that $\left(A_{22},A_{12}\right)$ is also observable or detectable. Therefore, for state ${\bar{x}}_{2}$, a full-order observer can be designed as:

(13) $${\hat{\bar{x}}}_{2}\left(t+1\right)=\left[{\bar{A}}_{22_{\sigma \left(t\right)}}-{\bar{L}}_{\sigma \left(t\right)}{\bar{A}}_{12_{\sigma \left(t\right)}}\right]{\bar{x}}_{2}\left(t\right)+{\bar{L}}_{\sigma \left(t\right)}w\left(t\right)+\bar{u}\left(t\right)$$

Combining (11) with (13), the following expression is constructed:

(14) $${\hat{\bar{x}}}_{2}\left(t+1\right)=\left[{\bar{A}}_{22_{\sigma \left(t\right)}}-{\bar{L}}_{\sigma \left(t\right)}{\bar{A}}_{12_{\sigma \left(t\right)}}\right]{\bar{x}}_{2}\left(t\right)+{\bar{L}}_{\sigma \left(t\right)}\Psi +{\bar{A}}_{21_{\sigma \left(t\right)}}y\left(t\right)+{\bar{B}}_{2}u\left(t\right)+{\bar{D}}_{2_{\sigma \left(t\right)}}v\left(t\right)$$

where $\Psi =y(t+1)-{\bar{A}}_{11_{\sigma \left(t\right)}}y\left(t\right)-{\bar{B}}_{1}u\left(t\right)-{\bar{D}}_{1_{\sigma \left(t\right)}}v\left(t\right)$.

Note that $z={\hat{\bar{x}}}_{2}-{\bar{L}}_{\sigma }y$, so we obtain

(15) $$\begin{array}{ll} z\left(t+1\right) & ={\hat{\bar{x}}}_{2}\left(t+1\right)-{\bar{L}}_{\sigma \left(t\right)}y\left(t+1\right)\\ & =\left[{\bar{A}}_{22_{\sigma \left(t\right)}}-{\bar{L}}_{\sigma \left(t\right)}{\bar{A}}_{12_{\sigma \left(t\right)}}\right]{\bar{x}}_{2}\left(t\right)+\left({\bar{A}}_{21_{\sigma \left(t\right)}}-{\bar{L}}_{\sigma \left(t\right)}{\bar{A}}_{11_{\sigma \left(t\right)}}\right)y\left(t\right)\\ & \begin{array}{l} +\left({\bar{B}}_{2}-{\bar{L}}_{\sigma \left(t\right)}{\bar{B}}_{1}\right)u\left(t\right)+\left({\bar{D}}_{2_{\sigma \left(t\right)}}-{\bar{L}}_{\sigma \left(t\right)}{\bar{D}}_{1_{\sigma \left(t\right)}}\right)v\left(t\right)\\ =F_{\sigma \left(t\right)}z\left(t\right)+G_{\sigma \left(t\right)}y\left(t\right)+Hu\left(t\right)+J_{\sigma \left(t\right)}v\left(t\right) \end{array} \end{array}$$

where

$$\{\begin{array}{l} F_{\sigma \left(t\right)}=A_{22_{\sigma \left(t\right)}}-L_{\sigma \left(t\right)}A_{12_{\sigma \left(t\right)}}\\ G_{\sigma \left(t\right)}=F_{\sigma \left(t\right)}L_{\sigma \left(t\right)}+A_{21_{\sigma \left(t\right)}}-L_{\sigma \left(t\right)}A_{11_{\sigma \left(t\right)}}\\ H_{\sigma \left(t\right)}=B_{2_{\sigma \left(t\right)}}-L_{\sigma \left(t\right)}B_{1_{\sigma \left(t\right)}}\\ J_{\sigma \left(t\right)}=D_{2_{\sigma \left(t\right)}}-L_{\sigma \left(t\right)}D_{1_{\sigma \left(t\right)}} \end{array}$$

The traffic states of $\hat{\bar{x_{2}}}$ can be reconstructed by

(16) $$\hat{\bar{x_{2}}}\left(t\right)=z\left(t\right)+{\bar{L}}_{\sigma \left(t\right)}y\left(t\right)$$

where ${\bar{L}}_{\sigma \left(t\right)}$ is the feedback matrix of the state observer.

The reconstructed traffic states ${\bar{x}}_{1}$ and ${\bar{x}}_{2}$ can be denoted as

(17) $$\{\begin{array}{l} \begin{array}{l} \begin{array}{l} \begin{array}{l} {\hat{\bar{x}}}_{1}={\bar{x}}_{1}=y \end{array} \end{array} \end{array}\\ {\hat{\bar{x}}}_{2}=z+L_{\sigma }y \end{array}$$

Correspondingly, the vehicle density estimates are obtained in the following equation:

(18) $$\hat{\bar{x}}=\left[\begin{array}{c} {\hat{\bar{x}}}_{1}\\ {\hat{\bar{x}}}_{2} \end{array}\right]=\left[\begin{array}{c} y\\ z+L_{\sigma }y \end{array}\right]$$

With $\bar{x}=Px$ being held to be true, we obtain $x=P^{-1}\bar{x}=Q\bar{x}$ and $\hat{x}=Q\hat{\bar{x}}$. This further leads to

(19) $$\hat{x}=\left[\begin{array}{cc} Q_{1} & Q_{2} \end{array}\right]\left[\begin{array}{c} y\\ z+L_{\sigma }y \end{array}\right]=Q_{1}y+Q_{2}\left(z+L_{\sigma }y\right)$$

**Figure 2:**
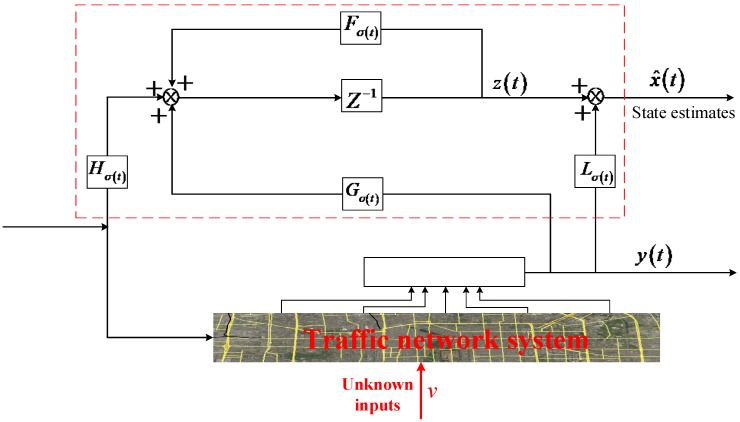
The structure of the reduced-order unknown-input state observer.

Finally, the reduced-order state observer can be derived according to (9). Figure 2 illustrates the structure of the reduced-order unknown-input state observer.

### 2.3. Estimation of Observer Parameters

Further to establishing the structure of the reduced-order unknown-input state observer as (9), the parameters of its unknown input vector v(t) need to be estimated for real-world implementation. To initiate this process, the matrix $L_{\sigma \left(t\right)}$ in (16) can be computed by imposing the following condition:

(20) $$J_{\sigma \left(t\right)}=D_{2_{\sigma \left(t\right)}}-L_{\sigma \left(t\right)}D_{1_{\sigma \left(t\right)}}=0$$

Meanwhile, the matrix $F_{\sigma \left(t\right)}$ must possess Schur stability, so that the existence of the state observer as described by Expression (9) is guaranteed. This implies that the matrix $A_{22_{\sigma \left(t\right)}}-L_{\sigma \left(t\right)}A_{12_{\sigma \left(t\right)}}$ has stable eigenvalues. Also, it is essential to compute the feedback matrix $L_{\sigma \left(t\right)}$ in order to determine the matrix ${\bar{D}}_{\sigma \left(t\right)}$. The procedure for deriving the matrix $L_{\sigma \left(t\right)}$ is given below.

First, with the above assumptions in place, the following conditions are satisfied:

(21) $$\mathrm{rank}D_{\sigma }=\mathrm{rank}(CD_{\sigma })=\mathrm{rank}D_{1_{\sigma }}=p$$

Then, the matrix $L_{\sigma \left(t\right)}$ can be computed by combining (20) and (21):

(22) $$L_{\sigma }=D_{2_{\sigma }}D_{1_{\sigma }}^{+}+K_{\sigma }(I_{q}-D_{1_{\sigma }}D_{1_{\sigma }}^{+})$$

where $D_{1_{\sigma \left(t\right)}}^{+}$ is the generalized inverse of $D_{1_{\sigma \left(t\right)}}$.

Because the matrix $D_{1_{\sigma }}$ is of full column rank, the matrix $D_{1_{\sigma \left(t\right)}}^{+}$ can be calculated by:

(23) $$D_{1_{\sigma }}^{+}={\left(D_{1_{\sigma }}^{T}D_{1_{\sigma }}\right)}^{-1}D_{1_{\sigma }}^{T},\;\;\forall K\in R^{(n-q)\times q}$$

Also, there exists an orthogonal matrix $S_{\sigma }$ with the following conditions satisfied:

(24) $$\{\begin{array}{l} S_{\sigma }D_{1_{\sigma }}=\left[\begin{array}{c} {\bar{D}}_{1_{\sigma }}\\ 0 \end{array}\right]\\ S_{\sigma }A_{12_{\sigma }}=\left[\begin{array}{c} {\bar{A}}_{12_{\sigma }}^{1}\\ {\bar{A}}_{12_{\sigma }}^{2} \end{array}\right]\\ K_{\sigma }S_{\sigma }^{T}=\left[\begin{array}{cc} {\bar{K}}_{1_{\sigma }} & {\bar{K}}_{2_{\sigma }} \end{array}\right] \end{array}$$

where ${\bar{D}}_{1_{\sigma }}\in R^{p\times p}$ is a nonsingular matrix, ${\bar{A}}_{12_{\sigma }}^{1}\in R^{p\times 1}$, ${\bar{K}}_{1_{\sigma }}\in R^{1\times p}$.

Next, the matrices $F_{\sigma }$, $G_{\sigma }$, and $H_{\sigma }$ can be computed by:

(25) $$\begin{array}{ll} F_{\sigma } & =A_{22_{\sigma }}-L_{\sigma }A_{12_{\sigma }}\\ & =A_{22_{\sigma }}-D_{2_{\sigma }}D_{1_{\sigma }}^{+}A_{12_{\sigma }}+K_{\sigma }(I_{q}-D_{1_{\sigma }}D_{1_{\sigma }}^{+})A_{12_{\sigma }}\\ & =A_{22_{\sigma }}-D_{2_{\sigma }}{\bar{D}}_{1_{\sigma }}^{-1}{\bar{A}}_{12_{\sigma }}^{1}-{\bar{K}}_{2_{\sigma }}{\bar{A}}_{12_{\sigma }}^{2}\\ & =F_{1_{\sigma }}-{\bar{K}}_{2_{\sigma }}{\bar{A}}_{12_{\sigma }}^{2} \end{array}$$

(26) $$G_{\sigma \left(t\right)}=F_{\sigma \left(t\right)}{\bar{L}}_{\sigma \left(t\right)}+{\bar{A}}_{21_{\sigma \left(t\right)}}-{\bar{L}}_{\sigma \left(t\right)}{\bar{A}}_{11_{\sigma \left(t\right)}}$$

(27) $$H_{\sigma \left(t\right)}={\bar{B}}_{2}-{\bar{L}}_{\sigma \left(t\right)}{\bar{B}}_{1}$$

### 2.4. Design of the State Observer

After developing the procedure for estimating the parameters for the reduced-order unknown-input state observer, the subsequent effort is centered on proposing a design procedure of the state observer for switched systems. The design comprises a generic process with the following steps:

*Step 1*: Compute matrices $A_{\sigma \left(t\right)}$, B, and $D_{\sigma \left(t\right)}$ and construct output matrix C using data collected by traffic-counting sensors for the preparation of traffic flow modeling;

*Step 2*: Verify the validity of the conditions $\mathrm{rank}C=q,\mathrm{rank}D_{\sigma }=\mathrm{rank}\left(CD_{\sigma }\right)=q,q\geq p$ and iteratively reconfigure the matrices C and $D_{\sigma }$ until the above conditions are satisfied;

*Step 3*: Estimate the state transformation matrix $P≜\left[\begin{array}{c} C\\ R \end{array}\right]$; let $\bar{x}=P^{-1}x$ and calculate the values of (6);

*Step 4*: Determine the feedback matrix $L_{\sigma }=D_{2_{\sigma }}D_{1_{\sigma }}^{+}+K\left(I_{q}-D_{1_{\sigma }}D_{1_{\sigma }}^{+}\right)$;

*Step 5*: Develop an (n×n) orthogonal matrix $S_{\sigma }$ that satisfies the conditions in (24);

*Step 6*: Generate the matrix ${\bar{K}}_{2_{\sigma }}$ to satisfy Schur stability for $F_{\sigma }$;

*Step 7*: Derive the matrices $G_{\sigma }$ and $H_{\sigma }$;

*Step 8*: Establish the reduced-order unknown-input state observer for switched systems in accordance with (9).

## 3. Case Study: Beijing Jingtong Freeway

In this section, an experiment example will be presented to demonstrate the validity and the practicability of the proposed approach by applying the designed state-jump observer to the Beijing Jingtong freeway. The selected road section is approximately 3.5 km long and is comprises three lanes. In accordance with the segment partition rules mentioned in reference [17], the road section was divided into 10 cells, as shown in Table 1.

### 3.1.  Data Collection and Processing

**Figure 3:**
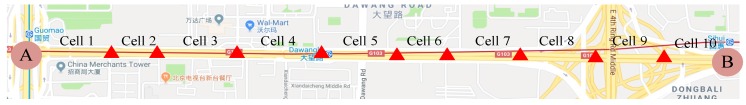
Illustration of the Jingtong freeway segment for the methodology application.

Figure 3 presents the segment of Jingtong Freeway, Beijing, China used for methodology application, particularly the design of an unknown-input state observer for analyzing traffic states to reconstruct vehicle densities essential to traffic flow modeling. The west–east directional freeway segment is labeled as segment AB and encompasses four on-ramps and four off-ramps, respectively labeled in ascending order from 1 to 4. The directional segment AB is partitioned into 10 cells marked from 1 to 10 correspondingly. Table 1 and Table 2 list the details of the cell lengths and pertinent parameters of vehicles’ operational characteristics.

### 3.2. Analysis Results

**Figure 4:**
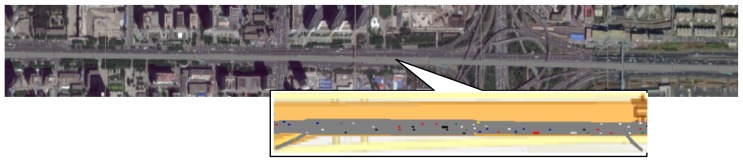
Topological representation of the Jingtong freeway segment in the VISSIM model.

Figure 4 depicts a VISSIM-based simulation model developed for the directional segment AB to generate simulated traffic stream data [42]. The simulation execution period was 3 h in a typical weekday from noon to 3:00 p.m., with a data reporting interval of 5 s. Virtual traffic-counting sensors were placed in each cell to collect data on vehicle densities used to verify the accuracy and precision of traffic state estimates from the reduced-order unknown-input state observer. In addition, virtual traffic-counting sensors were installed in the on-ramp Section 2 and Section 3 to collect traffic data as known inputs. Conversely, on-ramp Section 1 and Section 4 were not equipped with traffic-counting sensors and could be treated as roadway sections with unknown inputs.

**Remark** **4.** *Although many traffic simulation software have been developed for traffic system modelling, VISSIM is one of the most practical traffic simulation software to model urban traffic and public transit operations. Compared with other traffic simulation software, such as SParamics, VISSIM reserves many data interfaces, which enables users to easily redevelop the model of interest in accordance with their own needs by incorporating some new algorithms. As a result, a VISSIM model can be modified to continue previous research, which provides significant opportunities for sustainable and comprehensive traffic simulation research.*

**Figure 5:**
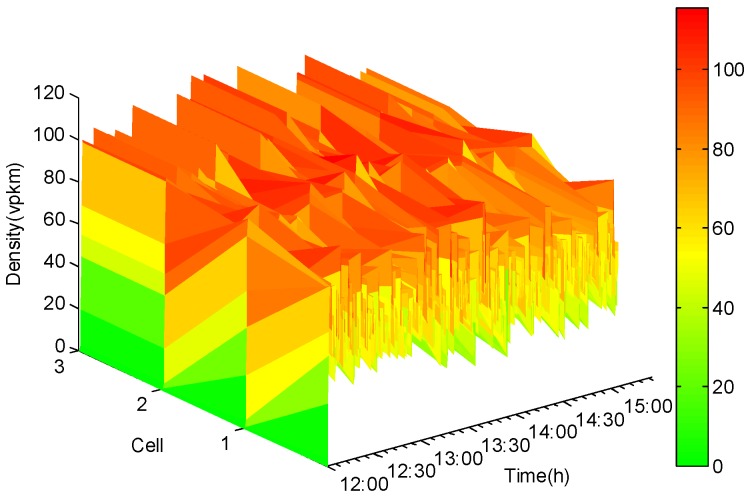
Simulated data for vehicle densities.

**Figure 6:**
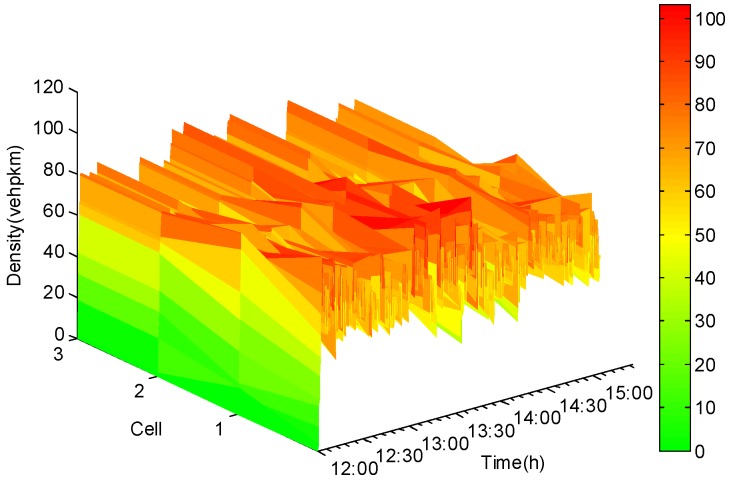
Estimated vehicle densities.

As sufficient and necessary conditions for the existence of the unknown-input state observer, the pair $\left(A_{\sigma },C\right)$ must be observable or detectable. In this respect, traffic-counting sensors were installed in cells 2, 3, 4, 5, 6, 8, 9, and 10, which facilitated collecting data on vehicle densities that were subsequently used to design the observer. Therefore, the output matrix *C* = [*c_i,j_*] was a 7 × 10 matrix, and $c_{1,2}=c_{2,3}=c_{3,4}=c_{4,6}=c_{5,8}=c_{6,9}=c_{7,10}=1$, with all other entries of the matrix set to zero. With virtual traffic-counting sensors installed in cells 2, 3, 4, 5, 6, 8, 9, and 10, data on vehicle densities of those cells could be directly collected. For the remaining cells including cells 1, 5, 7 without virtual sensors, vehicle densities associated with them needed to be derived based on traffic states estimated by the reduced-order unknown-input state observer. Figure 5 and Figure 6 show the simulated and estimated vehicle densities for multiple cells evolving over different time points of the analysis. Using a color-coding mechanism of green, yellowand red, vehicle densities would increase from green to yellow and then to red, representing free-flow to capacity and then to congested traffic stream conditions. As the red color becomes darker, it shows more severe traffic congestion.

**Figure 7:**
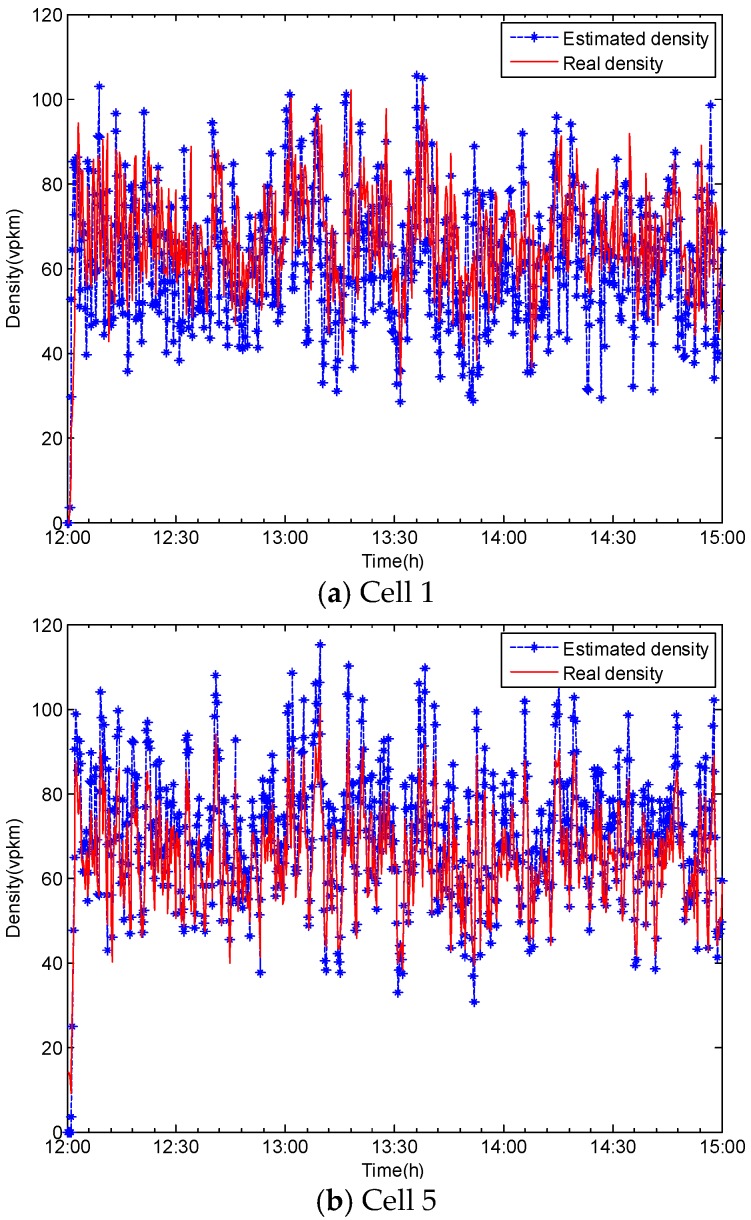

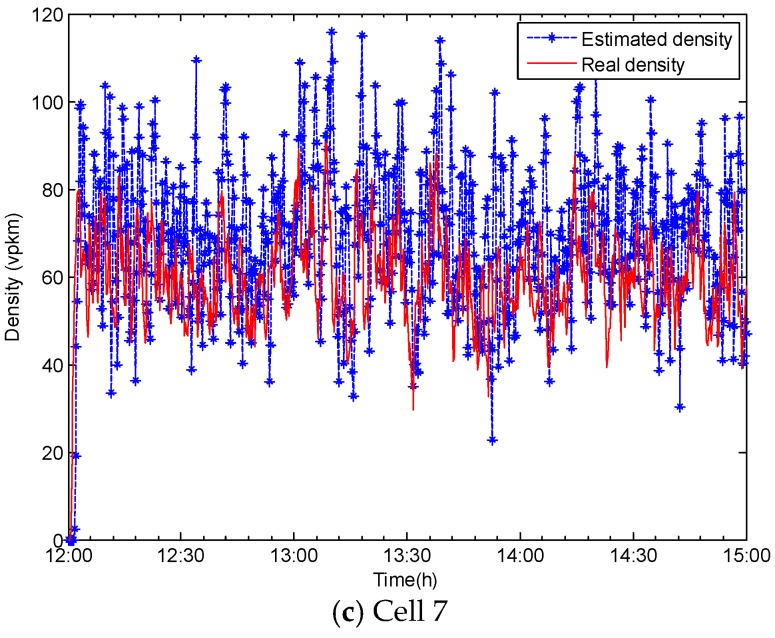
Comparison of simulated and estimated vehicle densities for (**a**) Cell 1, (**b**) Cell 5, and (**c**) Cell 7.

Further, vehicle densities for cells 1, 5, and 7 were reconstructed using traffic state estimates by the reduced-order unknown-input state observer, as shown in Figure 7.

Vehicle densities could be reconstructed by the designed reduced-order unknown-input state observer withmodest accuracy. Compared with the results of the known-input observer [11], the estimation needs to be more precise. Once this issue gets resolved, the proposed methodology will become highly practical for deployment to a large traffic network.

Congested road segments could be further identified from the estimation results. Thus, driving-route planning could be optimized effectively for GPS systems, greatly enhancing travel efficiency.

**Remark** **5.** *It is well known that for the most reliable validation, the proposed model should be evaluated using real-world traffic data. However, it is always difficult to obtain real-world data. We have tried our best to measure traffic data from the real world and will present the analysis result in our future work. Nevertheless, computer simulation has already been recognized as an effective tool to verify various theoretical models; in this study, the effectiveness of the proposed method was verified using simulated data.*

**Remark** **6.** *In this study, only one street was considered in the method validation. It is very important to consider the influence of adjunction streets to analyze the effects of other cells and streets on the traffic network. To this end, as a first step, this study introduced on-ramp traffic into the analyzed street to investigate its effect on the main road.*

**Figure 8:**
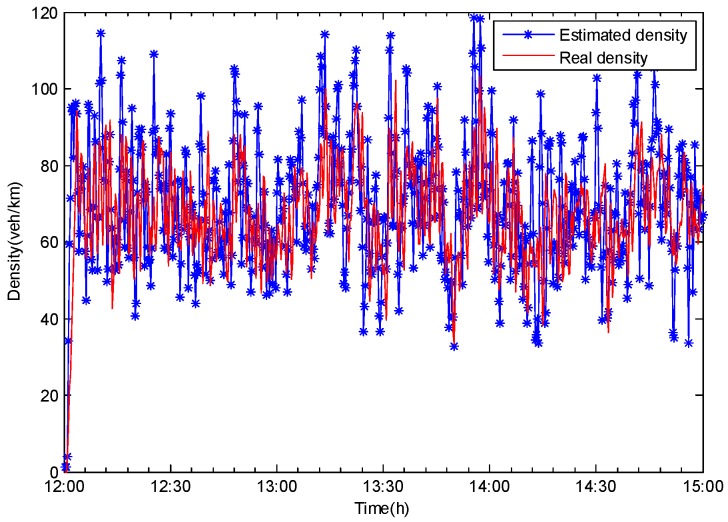
Cell 1 with on-ramp traffic.

In our case, on-ramp traffic flowed into cell 1. The on-ramp traffic volume was 10% of the cell 1 traffic. The prediction results for cell re ias shown in Figure 8. As can be seen, the proposed model was still effective for traffic density prediction. However, compared to Figure 7a, because the traffic flow on the main road was affected by the on-ramp vehicles, the estimated accuracy of vehicle density determination was reduced by 15%. As a result, it was crucial to consider the influence of other streets on traffic flow and develop related observer functions to reduce it and improve the accuracy of the prediction model. This coupled-street issue will be addressed in our next work.

## 4. Conclusions

Based on the hybrid dynamic traffic system with unknown inputs, a switched unknown-input state observer was designed, and the issues of vehicle density estimation and congestion identification were investigated. We showed that the unknown-inputsobserver was able to reconstruct the vehicle densities of road sections which were not equipped with traffic sensors. This strategy for vehicle density estimation was applied to The Beijing Jingtong freeway. Experimental results demonstrated that the estimated densities matched the actual densities reasonably well, and thus congestion can be identified effectively using ths model.

However, in this study, only simulation data obtained by VISSIM were used to verify the performance of the observer, thus the results have some limitations. Meanwhile, the design of the model parameters did not consider the coupled-street effect. In future work, we will choose a practical road network and collect real data to evaluate and optimize the model parameters. The coupled-street issue will also be addressed.

## Figures and Tables

**Table 1:** Cell length.

Cell Number	Length (m)	Cell Number	Length (m)
1	300	6	275
2	160	7	435
3	460	8	400
4	430	9	450
5	400	10	406

**Table 2:** Cell-specific parameters of vehicles’ operational characteristics.

Cell Number	*V* (km/h)	*W* (km/h)	*C* (veh/h)	$\rho^{0}\;(\mathrm{veh}/\mathrm{km})$	$\rho^{m}\;(\mathrm{veh}/\mathrm{km})$
1–10	65	20	2800	46	185
